# Reversal of Hartmann's Procedure: Evaluating Outcomes of Single-Port Laparoscopic Approach Versus Conventional Approach

**DOI:** 10.7759/cureus.11916

**Published:** 2020-12-05

**Authors:** Ashley A Arnold, Vanessa May, Saruja Nanthakumaran, Sukrut Pagad, Manoj R Somagutta, Saijanakan Sridharan, Bilal Haider Malik

**Affiliations:** 1 Surgery, California Institute of Behavioral Neurosciences and Psychology, Fairfield, USA; 2 Research, California Institute of Behavioral Neurosciences and Psychology, Fairfield, USA; 3 Psychiatry, California Institute of Behavioral Neurosciences and Psychology, Fairfield, USA; 4 Internal Medicine, California Institute of Behavioral Neurosciences and Psychology, Fairfield, USA

**Keywords:** single-port laparoscopic surgery, single-incision laparoscopic surgery, reversal of hartmann's procedure, postoperative outcome hartmann reversal, laparoscopic reversal hartmann's procedure

## Abstract

Bowel restoration following Hartmann's procedure (HP) remains a topic of discussion and innovation. This article seeks to highlight and analyze the outcomes of conventional reversal approaches such as open surgery (OS) and conventional laparoscopic (CL) to single-port laparoscopic reversal (SPLR) approach to evaluate whether SPLR is a feasible alternative to the OS or CL approach. A PubMed search using keywords yielded 5,750 articles. After applying the inclusion/exclusion criteria, 40 articles of relevance were reviewed, and endpoints considered. These included 13 systematic reviews and 27 observational reviews, three of which identified themselves as retrospective or comparative studies. The analysis showed overwhelming support for CL over OS as a choice for HP reversal. Studies comparing SPLR to CL showed SPLR to be a safe and feasible alternative, given its significantly shorter operating times, hospitalization times, and complication rates.

## Introduction and background

The first description of Hartmann's procedure (HP) was in 1921, introduced by Henry Albert Hartmann, a French surgeon, and although its primary intention was rectal carcinoma resection, this approach was taken in various diverticular diseases [1-3]. HP involves a sigmoidectomy with end colostomy resulting in a closed anorectal stump [4].

Since then, colostomy reversal has been a topic of discussion aimed at reducing morbidity and mortality rates [5,6]. While conventional approaches of Hartmann's reversal [HR] such as open surgery (OS) and conventional laparoscopic (CL) surgery have been preferred, laparoscopic approaches have become increasingly favored by many for its reduced postoperative morbidity, wound infection, length of stay, and ileus formation. Operative access trauma by laparotomy can also be minimized.

Since first being reported in 2011, the use of a single-port laparoscopic reversal (SPLR) technique has been explored. SPLR has been found safe and feasible via the colostomy site and maybe a considered surgical option for experienced surgeons in selected patients [7]. Thambi et al. noted that compared to OS and CL, SPLR had shorter operating times and hospitalization, with no discernible difference in morbidity [8]. This article seeks to highlight the outcomes between CL and SPLR techniques to evaluate whether SPLR is a feasible alternative to the CL approach.

Methods

Literature was searched in PubMed with strategies based on regular and medical subject headings (MeSH) keywords for data collection. Total results were filtered using inclusion and exclusion criteria, yielding selected results which can be seen in Table 1.

**Table 1:** Total results of MeSH keywords and selected results based on inclusion and exclusion criteria HP: Hartmann's procedure, MeSH: medical subject heading, HR: Hartmann's reversal.

MeSH Keywords	Database	Total Results	Selected Results
Single-port laparoscopic surgery	PubMed	2966	631
Single-incision laparoscopic surgery	PubMed	2060	438
Reversal of HP	PubMed	468	97
Postoperative outcome of HR	PubMed	108	39
Laparoscopic reversal of HP	PubMed	148	44
Total		5750	1249

Studies were selected after applying the following inclusion/exclusion criteria. Inclusion criteria were applied in the following order: (i) literature published in the last five years; (ii) literature published in the English language; (iii) human studies; (iv) all full-text papers.

Results

Of the 5750 literature papers found, a total of 4501 were excluded based on inclusion/exclusion criteria, leaving 1249 selected results. After analysis, a total of 1209, according to Table 1 keyword searches, were removed due to one or more of the following reasons. They did not involve or specify the procedure of interest (that is, those that did not include any information on HP or reversal techniques). Literature that was based on post-procedure repairs such as laparoscopic repair of perforation, fistula, or anastomosis leak post-HP were excluded as well as comments on previous publications, case reports, meta-analysis, and multicenter studies. Duplicate papers and those that were unable to be accessed were also excluded. Finally, 40 publications in PubMed were reviewed, which included 27 observational studies, among which three were identified as retrospective/comparative studies and 13 systematic reviews.

## Review

HP was first developed with the intent of treating rectosigmoid carcinoma by Henry Hartmann and involves rectosigmoid colon resection with the creation of a colostomy. It has a variety of indications, including complicated diverticulitis and less commonly volvulus, ischemia, and perforation [9]. Studies report a significant reduction in quality of life and gradual social isolation in HP patients [3,7]. Research into the reversal of HP has therefore been of particular importance in qualified patients, intending to restore continuity and lessen not only the physical but also the psychological challenges associated with colostomy [10-12]. However, this procedure is highly technical and poses many difficulties such as wound infection and ileus [5].

Discussion

Conventional Laparoscopy 

**Figure 1:**
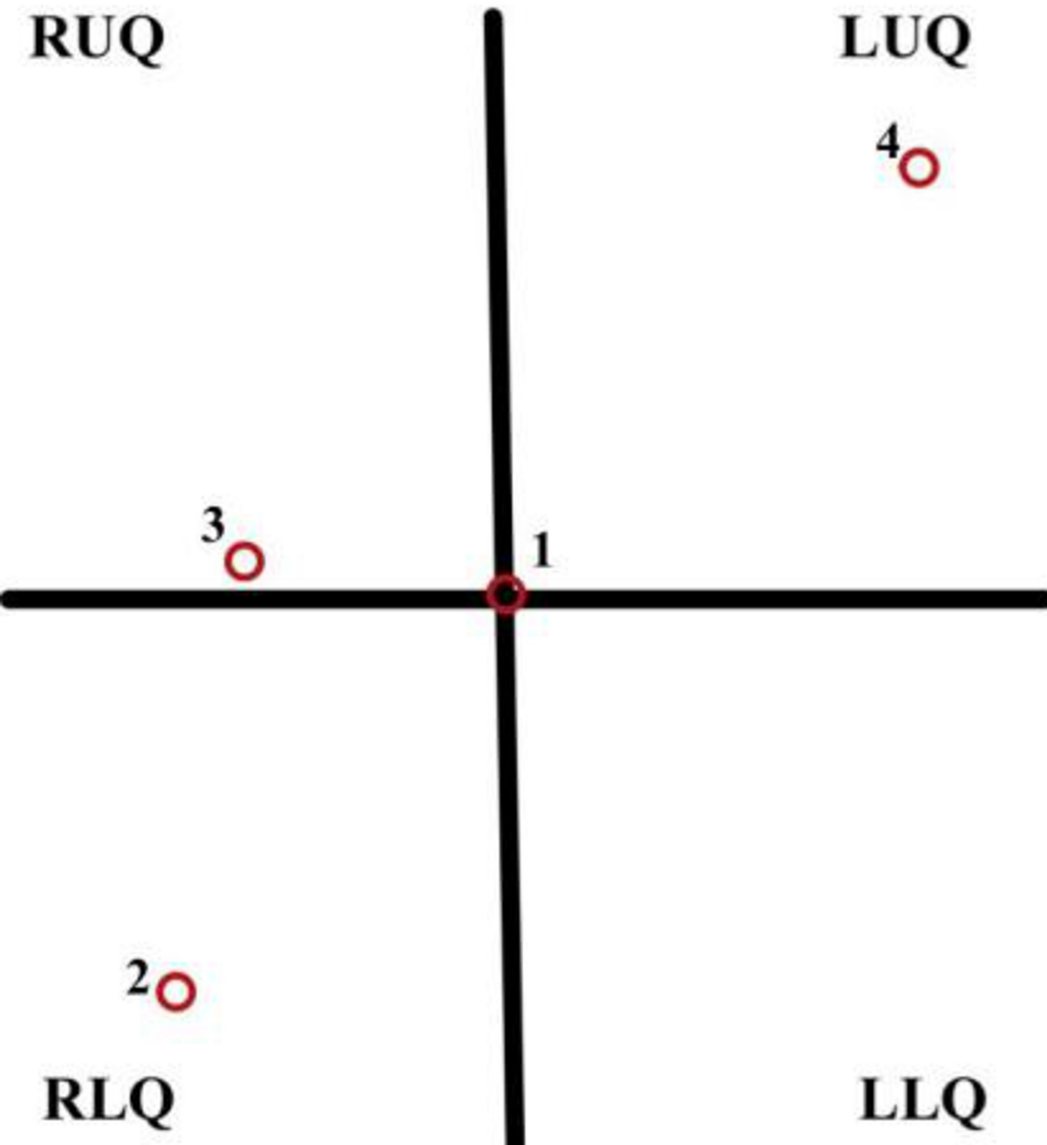
Trocar configuration of the patient’s abdomen during CL - the multi-port laparoscopic reversal of HP (1) 12 mm trocar at umbilicus, (2) 12 mm trocar along RLQ, (3) 5 mm trocar along with the right superior paramedian position, and (4) 5 mm trocar along LUQ. RUQ: right upper quadrant, RLQ: right lower quadrant, LUQ: left upper quadrant, LRQ: left lower quadrant, CL: conventional laparoscopy, HP: Hartmann’s procedure.

Open surgical (OS) reversal has also been associated with a considerable risk of morbidity and mortality. Thus, in an attempt to reduce these risks, research and development of multiport laparoscopic reversal were undertaken [13], which is now considered a conventional alternative approach (CL) to this procedure. CL involves the patient, in a modified lithotomy position with incisions made and trocars placed at the umbilicus, right lower quadrant, right superior paramedian position, and in addition may be placed in the left upper quadrant, depending on the presence of intra-abdominal adhesions. A circular-end-to-end anastomosis (CEEA) stapler is inserted transanally. After successful anastomosis, port sites are closed using non-absorbable sutures using the ostomy site approach and involves delayed closure, packing, and secondary intention [13-15]. Studies show that patients undergoing laparoscopic colostomy reversal have shown a reduced risk of complication compared to those who underwent OS [1,3,13,16-18]. The locations of the trocar placement during the CL procedure can be seen clearly in Figure 1.

Single-Port Laparoscopic Reversal

**Figure 2:**
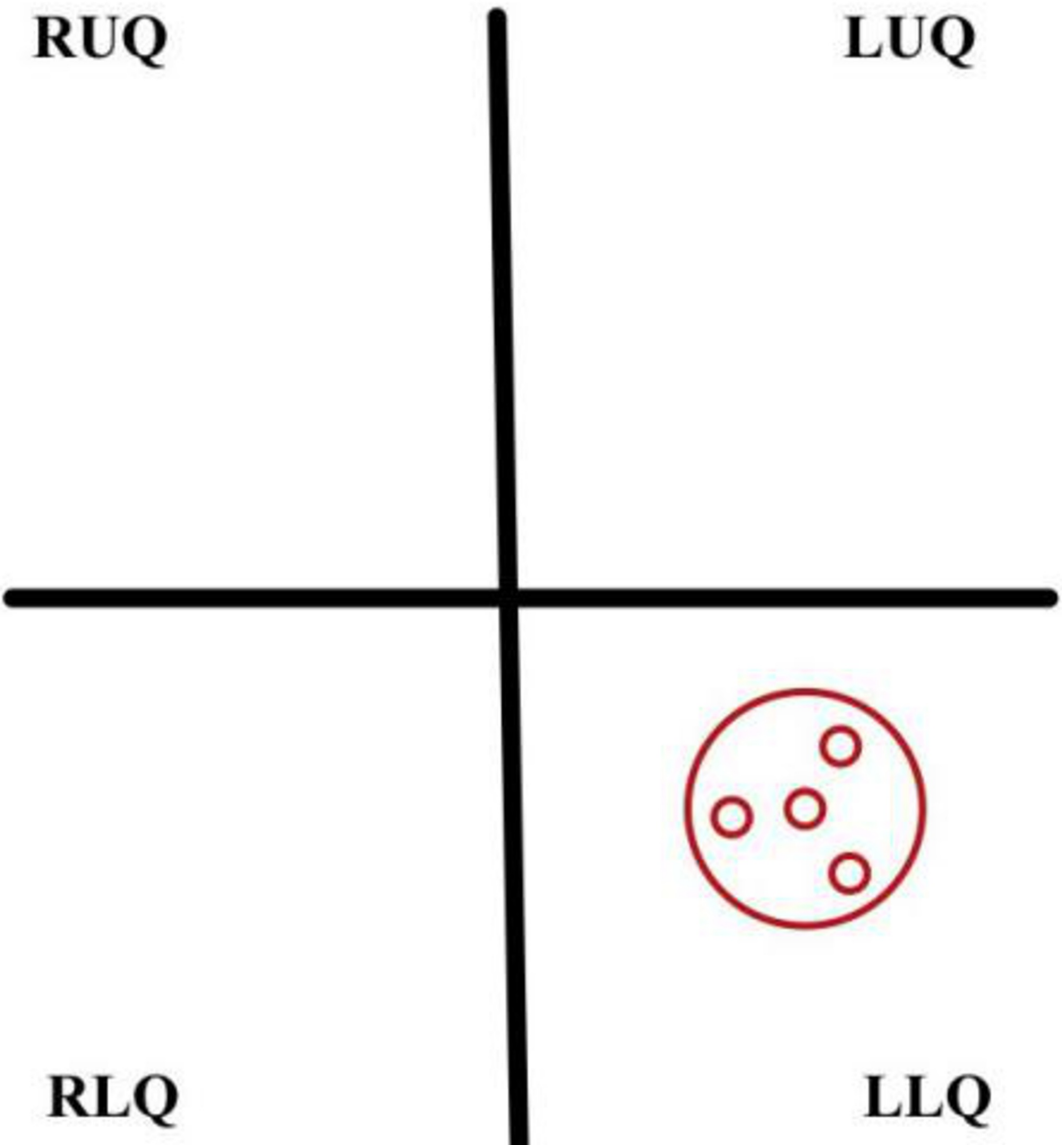
Single-port access device placement for SPLR through fascia defect at the stoma site RUQ: right upper quadrant, RLQ: right lower quadrant, LUQ: left upper quadrant, LRQ: left lower quadrant, SPLR: single-port laparoscopic reversal.

Although the CL technique for restoration of intestinal continuity has gained popularity due to its advantageous nature, it involves extensive adhesiolysis in which may increase the risk of paralytic ileus or bowel lacerations [19]. SPLR uses the existing colostomy site as the entry point. The elimination of multiple entry points decreases trauma and morbidities, such as infection. This technique involves the patient being placed in a modified lithotomy position, followed by stoma excision and using a single-port access point; two trocars are used to dissect adhesions and ensure rectal stump mobility [7-8,13]. CEEA stapler is introduced transanally and carefully removed following anastomosis. Single-port is removed and closed using non-absorbable sutures with ostomy site undergoing delayed closure, packing, and secondary intention. In contrast, some studies employ the use of absorbable sutures at the ostomy site and absorbable or non-absorbable for skin closure [7]. The location of the SPLR trocar placement during the procedure can be seen clearly in Figure 2.

The characteristic finding among the literature reviewed suggested that the CL approach’s benefits outweigh that of OS in HR. A 2015 study consisting of 862 patients, with negligible differences, found that the 403 patients who underwent CL had a lesser incidence of wound infection, post-op ileus, and morbidity; however, there was no difference in operating time [1]. This differs from studies showing no statistical difference in demographic, comorbidity, operative time, blood loss, reoperation, and readmission between CL and OS-HR, except for shorter hospitalization stay [4]. A similar study conducted in 2017 comprising of 29 patients of similar characteristics, showed the only significant difference in outcome to be that of faster bowel function recovery in CL patients. This study concluded that there were no significant differences between OS and CL concerning mean operation time, length of hospital stay, post-op analgesic days, time of diet resumption, or complication rate [14,20]. These findings were supported by other literature, showing minimal significant differences in operative time and complication rates [21,22]; however, a 2016 study associated CL with significantly decreased estimated blood loss and concluded CL could be safely performed with better short-term outcomes [16].

However, literature that considered SPLR found it to be superior to the CL approach (i.e., multi-port laparoscopic reversal) [23,24]. A 2019 study showed that compared to OS and CL, SPLR resulted in significantly shorter operating times and hospitalization [8,13], with significantly fewer complications in the SPLR group vs. OS [14]. There were also no discernible differences in morbidity [8] and no recorded mortality [13]. There is, unfortunately, no long-term data outcomes for the patients in these studies.

When the SPLR technique was first introduced, limitations such as cost of technique and equipment were of concern. However, studies have stated that cost is comparable to that of four disposable laparoscopic ports and wound protection device. Considering shorter operation times and hospital stays, the overall cost of disposable items is most likely recovered.

The studies evaluated above were summarized highlighting the main points, with reference to patient outcomes post-CL or OS versus SPLR procedure, where applicable, and can be seen in Table 2.

**Table 2:** Summary of some studies evaluated for literature review CL: conventional laparoscopy, OS: open surgery, SPLR: single-port laparoscopic reversal.

Authors	Country	Study Design	Sample Size	SPLR	CL/OS	Main Points
Thambi et al. [8]	UK	Systematic review	106	56	34	Compared to OS and CL, SPLR has shorter operating times (146 minutes vs 211 minutes) and hospitalization (4 days SPLR vs 6 days CL, 7 days OS). There are no discernible differences in morbidity.
Clermonts et al. [13]	-	Systematic review	41	25	16	SPLR techniques show shorter operative times and significantly shorter hospital stay (4 days SPLR vs 16 days CL). The number of complications was significantly lower in the SPLR group (10 SPLR vs 33 CL). No recorded mortality.
Choi et al. [7]	Korea		23	22 successful		Procedure aborted in one patient. SPLR had a median operation time of 165 minutes (100-340 minutes) and 8 days post-op length of stay (4-31 days). The average time of resumption of oral intake was 3 days (1-16 days). No intraoperative complications. Four post-op complications, including one anastomotic leak.
Celentano et al. [1]	-	Systematic review	862	-	403 CL; 459 OS	CL had a lesser incidence of overall morbidity, time of flatus, wound infection, and postop ileus than OS. No significant difference in operating time.
Brathwaite et al. [4]	USA	Systematic review	81	-	19 CL; 62 OS	CL had a shorter length of hospitalization (5.7 days vs 7.9 days, P<0.01). Demographics, comorbidities, mean operative times, blood loss, reoperation, and readmission rates showed no statistically significant differences between the groups.
Kwak et al. [14]	South Korea	Comparative study	29	-	17 CL; 12 OS	CL showed faster bowel function recovery. CL and OS had a mean operation time of 212.5 minutes vs 251.8 minutes and time of diet resumption of 3.9 vs 6.2, respectively. Length of stay, post-op analgesic days, and complication rate showed no statistically significant differences.
Onder et al. [16]	USA	Comparative study	36	-	18 CL; 18 OS	CL is associated with significantly decreased estimated blood loss, faster bowel function restoration, and reduced hospital stay. No significant differences in operative time or complication rates.
Gavrila et al. [15]	Romania	-	9	-	5 CL; 2 OS 1 robotic	CL had a shorter average operating time, hospital stay, and bowel motility restoration time.
Pei et al. [17]	USA	Retrospective study	11,762	-	2423 CL; 9339 OS	CL had a shorter total length of hospital stay, operation time, and overall complication rates.
Giuseppe et al. [2]	Italy	Systematic review	20	-	19 CL successful	Procedure aborted in one patient. CL had an average operating time of 176 minutes (115-330 minutes). Bowel restoration occurred between 3-5 days with a mean length of stay of 7 days (4-11 days). There were no cases of anastomotic dehiscence, postop complications, or mortality. Late post-op complications of one incisional hernia occurred with no others reported in 3 years follow-up.

## Conclusions

The objective of this study was to determine the feasibility of SPLR as an alternative to conventional approaches such as OS or multi-port laparoscopic reversal (CL) of HP. The current literature concluded that SPLR is a safe and feasible therapeutic alternative to OS and CL, when performed by an experienced surgeon, with an acceptable morbidity and mortality rate and taking into consideration its difficulty level. Based on its advantages of significantly shorter operating times, hospitalization, and lower complication rates, it should be considered a treatment course for colostomy closure after HP. However, SPLR warrants further clinical trials and comparative investigations.
